# Theoretical study of the properties of X-ray diffraction moiré fringes. I. Corrigenda and addenda

**DOI:** 10.1107/S2053273319006557

**Published:** 2019-06-26

**Authors:** Jun-ichi Yoshimura

**Affiliations:** a Sakai 5-13-2-A322, Musashino-shi, Tokyo 180-0022, Japan

**Keywords:** diffraction moiré fringes, *Pendellösung* oscillation, phase jump, gap phase

## Abstract

Seven corrections are made and several supplementary equations are added to the article by Yoshimura [*Acta Cryst.* (2015), A**71**, 368–381].

On p. 371, left column, near the top, just after ‘the index (*i*) = (1, 2) … dispersion surface’, the following comment should be added: ‘the upper sine in equations (5*a*), (5*b*) refers to the case of *i* = 1, and the lower sine to the case of *i* = 2’. On p. 371, right column, near the bottom, just after ‘the indices (*i*, *j*) = (1, 2) …, respectively’, the following comment should be added: ‘the upper sign in equations (15*a*) to (15*d*) refers to the case of *j* = 1, and the lower sine to the case of *j* = 2’. Equations (14*b*), (14*c*) are incorrect. They must be corrected to
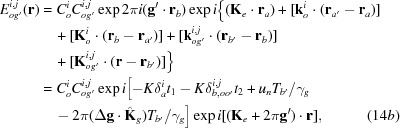


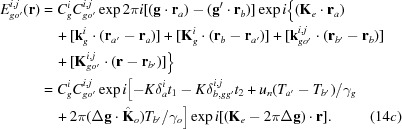



In these corrections, the last terms in the first exponential functions on the right-hand side in the second equations were corrected.

On p. 372, right column, an error is involved in equation (22*a*). It must be corrected to
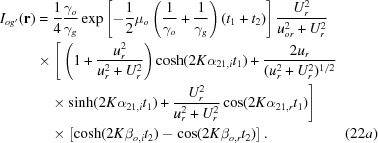



In this correction, the last term in the second bracket on the right-hand side was corrected. On p. 373, left column, equation (24*b*) is incorrect. It must be corrected to
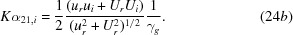



On p. 376, left column, the description of ‘*t*_gap_ = 0.024 mm’ is incorrect. It must be corrected to ‘*t*_gap_ = 0.24 mm’. The mentioned errors in equations (14*b*), (14*c*) and (24*b*) do not influence the calculation of equations (20) to (23*b*), since the correct expressions as above were used in deriving them. The mentioned error in equation (22*a)* does not influence the computations of the presented images and graphs in the paper, since the computations were all made correctly using the correct expression as mentioned above; the errors are only in the text.

As a supplement to the previous presentation of the equation for the diffracted – or *G* – wave image intensity 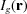 in equation (20), the equation for the corresponding transmitted – or *O* – wave image intensity 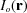 is added as in the following:
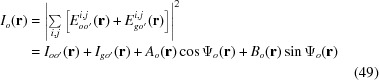
with
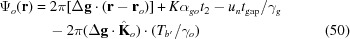





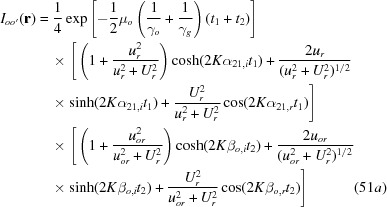


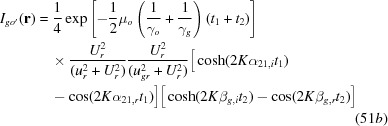


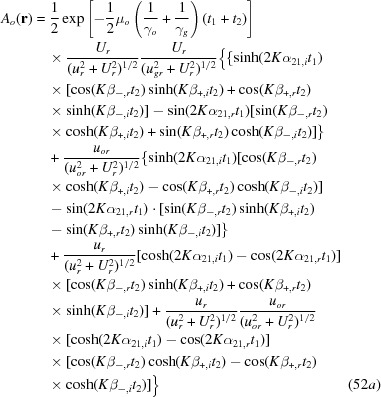


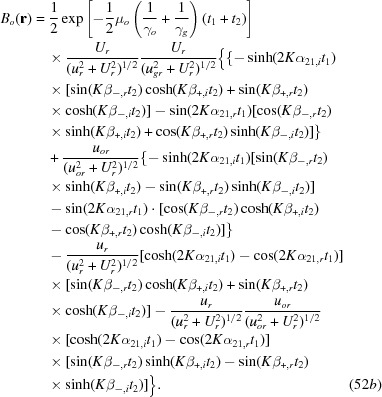



The numbering of the equations here is continued from the last equation (48) in the original paper (Yoshimura, 2015 ▸). 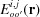 and 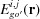 in equation (49) are as given in equations (14*a*), (14*c*), respectively.

Through similar calculations to those written in the right-hand side column on p. 373, the term of interference phase 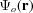 in equation (50) can be reduced to
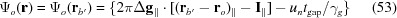
which is the same as 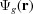 in equation (34) for the *G*-wave image intensity (here, the symbol || denotes the component parallel to the specimen surfaces). In the present case that 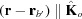, part of the first term and the fourth term in equation (50) cancel each other as follows:
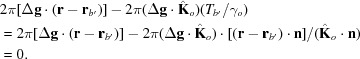



The moiré images of the *O*-wave in Figs. 14(*a*) and 14(*b*) and the curves concerned in Figs. 15(*a*) and 15(*b*) were computed using these equations (49), (51*a*)–(52*b*) and (53).
